# Supplementary material for: Differential immunoregulation by human surfactant protein A variants determines severity of SARS-CoV-2-induced lung disease
Source: Front Immunol. 2025 Apr 2;16:1462278. doi: 10.3389/fimmu.2025.1462278 (PMC12000003; doi:10.3389/fimmu.2025.1462278)
Supplement: Supplementary file 1 [file DataSheet1.pdf]

Differential Immunoregulation by Human Surfactant Protein A Variants Determines Severity of SARS-CoV-2-induced Lung Disease

Ikechukwu B. Jacob[1,2]; Akinwunmi O. Lawal[1,2]; Salma S. Mahmoud[1]; Emerson M. Kopsack[1]; Erin Reynolds[2]; Qinghe Meng[1]; Hongkuan Fan[3], Paul T. Massa[2,4]; Saravanan Thangamani[2]; Hongpeng Jia [5]; Guirong Wang [1,2 *]

[1]Department of Surgery, SUNY Upstate Medical University, Syracuse, NY, 13210, USA

[2]Department of Microbiology and Immunology, SUNY Upstate Medical University, Syracuse, NY, 13210, USA

[3]Department of Pathology and Laboratory Medicine, Medical University of South Carolina, Charleston SC 29425, USA

[4]Department of Neurology, SUNY Upstate Medical University, Syracuse, NY, 13210, USA

[5]Department of Surgery, Johns-Hopkins University, Baltimore, MD 21205, USA

Running title: Impact of Human SP-A Variants in SARS-CoV-2-Induced ALI

Corresponding author:
Guirong Wang, PhD (Dr. rer. Nat.)
Department of Surgery, UH Room 8715
SUNY Upstate Medical University
750 E Adams St,
Syracuse, NY, 13210, USA

Phone: 315-464-6283
Fax: 315-464-6236
E-mail: wangg@upstate.edu

Conflict of Interest Statement
The authors confirm that no competing interest exists.
Author Contributions: G.W., H.J., S.T., and I.B.J. contributed to the conception and study design. I.B.J., A.O. L., and E.R. performed ELISA and multiplex assays. I.B.J. E.R., S.T and A.O.L., S.S.M., E.M.K. and Q.M contributed to infectious virus titration and mouse challenge studies. I.B.J., P.T.M, H.J., H.F., and G.W. contributed to infection assays and data analysis. I.B.J. P.T.M., H.F., and G.W. drafted the first version of the manuscript. All authors read and approved the manuscript.

SUPPLEMENTARY INFORMATION

Mouse Models

Mice breeding and maintenance was carried out at SUNY Upstate Medical University animal core facility. The animals were housed in a temperature-controlled room at 22 °C under specific pathogen-free conditions. Mice between 8-24 weeks of age of both sexes were used in this study. The animal experiments were approved by SUNY Upstate Medical University Institutional Animal Care and Use Committee and conducted according to the National Institutes of Health and ARRIVE guidelines on the use of laboratory animals. The initial K18 (hACE2/mSP-A) mice were obtained from Jackson Laboratories (Bar Harbor, ME); these mice are susceptible to SARS-CoV-2 infection since they express the hACE2 transgene. To examine the role of human SP-A variants in the context of SARS-CoV-2 infection, we generated transgenic mice that carry both hACE2 and SP-A transgenes (double humanized transgenic or double-hTG) in which the hACE2 and individual single-gene variants of SP-A1 ($6A^2$ and $6A^4$) and SP-A2 ($1A^0$ and $1A^3$) are expressed and the mouse SP-A gene has been deleted by breeding with previously characterized hTG SP-A mice (1). To do this, we initially crossed K18 mice that are hemizygous for the hACE2 transgene with our SP-A knockout (KO) mice to remove the mSP-A gene. Genotyping was performed after each filial generation. Subsequently, we crossed the hACE2/SP-A KO mice with mice carrying individual variants of either SP-A1 ($6A^2$ or $6A^4$) or SP-A2 ($1A^0$ or $1A^3$) genes in order to generate a double-hTG mouse model with hACE2 and single-gene variants of human SP-A. We examined the presence of hACE2 and SP-A genes in the mice by PCR genotyping and analyzed SP-A expression in the lung by immunoblotting. The double-hTG mice (hACE2/$6A^2$ ($6A^2$), hACE2/$6A^4$ ($6A^4$), hACE2/$1A^0$ ($1A^0$) and hACE2/$1A^3$ ($1A^3$) alongside hACE2/SP-A KO (KO) and hACE2/mSP-A (K18) mice were subsequently used for SARS-CoV-2 (delta) challenge studies.

Mouse Infection and Sample Processing

SARS-CoV-2- and mock-challenged mice (6 groups: $6A^2$, $6A^4$, $1A^0$, $1A^3$, K18, and KO) were anesthetized with isoflurane and infected intranasally (i.n.) with 30 µl (15 µl/nose) of virus solution containing 1×10^3 PFU of SARS-CoV-2 in 1X MEM media. Control (Sham) mice were inoculated with 30 µl of 1X MEM. After viral infection, mice were observed daily for morbidity (body weight) and mortality (survival). Mice showing >25% decrease in their initial body weight were defined as reaching the experimental endpoint and euthanized. Mice were sacrificed by anesthesia and exsanguination on days 2, 4, and 6 post-infection (pi) to obtain lung samples for viral load analysis by RT-qPCR, immunohistochemistry (IHC), and plaque assay. Cytokine and gene-expression changes in the lung were analyzed, and systemic cytokines in sera were also determined.

Histopathological Analysis

Fixed lung tissues were embedded as previously described (2). 5 µm sections of individual tissue samples were cut and stained with Hematoxylin and Eosin (H&E) and then microscopically examined. Lung injury was scored using a 0-2 scale by counting the amount of neutrophils in the alveolar space, neutrophils in the interstitial space, presence of hyaline membranes, proteinaceous debris filling the airspaces, and alveolar septal thickening as described by (3). Histopathological analyses were blinded and carried out by two independent pathologists.

Transcriptomics Analyses

The expression of 84 innate and adaptive immune genes in the lungs of SARS-CoV-2 infected mice including Sham was determined using RT^2 Profiler™ PCR array kit (Cat#: PAMM-052ZC-24, Qiagen). Total RNA was isolated from homogenized lung tissues using the Quick-RNA extraction miniprep kit (# R1055 Zymo Research, CA, USA) following the manufacturer's instructions and RNA concentration and purity was determined by spectrophotometry using the nanodrop machine (Thermo Scientific). cDNA was synthesized from 500 ng of total RNA using the iScript Reverse Transcription

Supermix for RT-qPCR (Cat#: 1708841, Biorad). Real-time PCR was performed using the RT^2 Profiler^{TM} PCR array system following the manufacturer's recommendation in the AB StepOnePlus Detection System (Applied Biosystems, Foster City, CA). The levels of expression of the mRNA of each gene in the different groups were normalized to the housekeeping gene (*HSP90*). Data was exported into an Excel spreadsheet and analyzed with Qiagen's PCR analysis web-based software (GeneGlobe Data Analysis Center). Relative gene expression changes were calculated by the $2^{-(\text{average}\Delta\Delta Ct)}$ method. Fold change (FC) values are compared relative to the sham group or compared between two infected groups. Gene expression FCs are considered significant when FC > 2.0, p-value > 0.05).

Results

Extended Table 1: Primer List For RT-qPCR Validation

**Gene (Mouse)**	Forward primer (5'- 3')	Reverse primer (5'- 3')
**Il18 201/202**	AAGTGCCAGTGAACCCCAGACCA	CACAGAGAGGGTCACAGCCAGTCC
**MyD88 203/204**	TCCGACCGTGACGTCCT	ACCATGCGGCGACACC
**Irak1**	TCCACCAAGCAGTCAAGCC	AAACCACCCTCTCCAATCC
**NOD2**	GCTGTCTTGGGATGTGCT	GGATGAAGGGAGTGAGTGTC
**Gapdh**	CCAATACGGCCAAATCC	CCAATACGGCCAAATCCG
**Stat3**	GGGCATTTTTATGGCTTTCAAT	GTTAACCCAGGCACACAGACTTC
**Jak2**	AGGCGACGGGAACAAGATGT	AGGCCATTCCCATCTAGAGC

Extended Fig.1. Differential lung injury after SARS-CoV-2 infection of humanized mouse lines

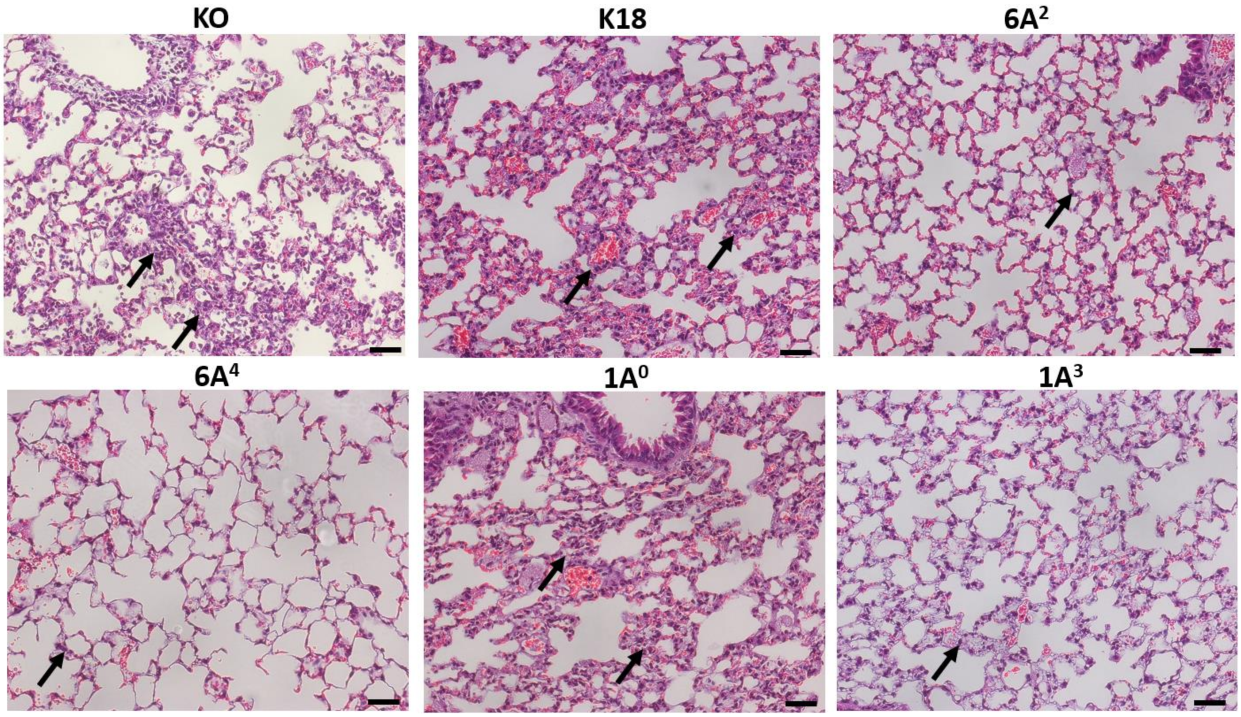

Extended Table 2

Differentially Expressed Genes (Infected KO vs Sham)		
**Gene Symbol**	**Fold Regulation**	**P-value**
Casp1	5.79	0.014756
Cd5	8.91	0.046393
Icam1	7.69	0.008094
Ifngr1	4.39	0.044389
Il17a	16.48	0.022416
Il18	19.19	0.004625

Il1r1	7.97	0.020679
Irak1	3.92	0.016144
Jak2	11.98	0.022278
Lyz2	5.63	0.019558
Myd88	6.40	0.010672
Nod2	17.93	0.023428
Stat1	6.28	0.017607
Stat3	12.27	0.014362
Actb	4.13	0.041631
B2m	6.17	0.030071
Gusb	3.54	0.023738
**Differentially Expressed Genes (Infected K18 vs Sham)**		
Ifnb1	28.76	0.000038
Il5	45.44	0.008027
Irf7	17.54	0.034547
Lyz2	4.57	0.002529
Mx1	283.34	0.012998
Nod2	11.91	0.009057
Stat3	5.19	0.026936
Tlr5	9.24	0.000889
Tlr7	13.55	0.019901
**Differentially Expressed Genes (Infected 6A^{2} vs Sham)**		
Casp1	5.76	0.035678
Ifng	118.07	0.016573
Il10	76.39	0.019744
Mx1	74.29	0.002725
Nfkb1	2.71	0.049131
Tlr3	3.99	0.026076
**Differentially Expressed Genes (Infected 6A^{4} vs Sham)**		
Il18	30.81	0.029020
Lyz2	12.26	0.000422
Stat1	4.30	0.013537
Tlr5	6.31	0.024732
B2m	16.77	0.038125
**Differentially Expressed Genes (Infected 1A^{0} vs Sham)**		
Ccl5	4.78	0.018746
Cd86	9.82	0.040562
Ddx58	4.14	0.007225
Il18	18.55	0.002340
Il1r1	5.43	0.036498
Irak1	4.72	0.001273

Ly96	5.71	0.034474
Mapk1	4.18	0.027030
Mapk8	4.44	0.011781
Nfkb1	3.82	0.033683
Stat3	6.64	0.042926
Tlr7	9.23	0.023362
Tlr8	41.35	0.012885
Gapdh	10.85	0.002370
Gusb	2.61	0.036120
**Differentially Expressed Genes (Infected 1A^3 vs Sham)**		
Ccl2	16.05	0.025912
Il10	21.73	0.049830
Il18	2.80	0.036129
Mx1	12.25	0.022571
Stat1	6.81	0.006183
**Differentially Expressed Genes (KO vs K18)**		
Icam1	7.61	0.018423
Myd88	13.16	0.004582
Il5	-4.54	0.026129
Mx1	-9.55	0.020986
**Differentially Expressed Genes (KO vs 6A^2)**		
Icam1	3.53	0.039577
Myd88	7.14	0.007947
Stat3	26.81	0.020250
Actb	3.84	0.045359
**Differentially Expressed Genes (KO vs 6A^4)**		
Csf2	37.43	0.003549
Ifna2	26.07	0.044254
Il17a	22.79	0.013839
Myd88	19.56	0.004296
Nod2	7.90	0.040216
Ticam1	8.54	0.020631
Tyk2	10.17	0.040750
Lyz2	-2.18	0.017263
**Differentially Expressed Genes (KO vs 1A^0)**		
Casp1	2.05	0.049517
Csf2	25.50	0.003665
Il17a	17.56	0.017414
**Differentially Expressed Genes (KO vs 1A^3)**		

Gata3	12.35	0.002657
Icam1	6.43	0.018416
Ifna2	22.71	0.032396
Ifngr1	4.73	0.009398
Il17a	7.60	0.021143
Il18	6.85	0.006584
Il1r1	17.28	0.014299
Irak1	4.97	0.009201
Jak2	6.44	0.032430
Lyz2	3.58	0.038574
Mapk1	2.28	0.022174
Myd88	14.54	0.016160
Nod2	9.60	0.028490
Stat3	6.00	0.028496
Stat4	10.29	0.000313
Ticam1	6.39	0.026506
Actb	3.48	0.044980
Gusb	3.87	0.032193
**Differentially Expressed Genes (K18 vs $6A^{2}$)**		
Mx1	3.81	0.034390
**Differentially Expressed Genes (K18 vs $6A^{4}$)**		
Csf2	29.29	0.006292
Ifnb1	61.15	0.000059
Il5	20.37	0.008441
Mx1	9.81	0.038784
Nod2	5.25	0.039805
Lyz2	-2.68	0.002301
**Differentially Expressed Genes (K18 vs $1A^{0}$)**		
Csf2	19.95	0.006556
Ifnb1	8.06	0.003828
Il5	7.32	0.016924
Mx1	8.69	0.025430
**Differentially Expressed Genes (K18 vs $1A^{3}$)**		
Ifnb1	20.78	0.000018
Il5	49.38	0.007167
Irf3	2.22	0.039937
Lyz2	2.90	0.015772
Mx1	23.13	0.014983
Nod2	6.38	0.013982
Tlr5	3.27	0.002488
Tlr7	26.55	0.012512

**Differentially Expressed Genes ($6A^2$ vs $6A^4$)**		
Cd86	4.78	0.031320
Lyz2	-8.85	0.021367
**Differentially Expressed Genes ($6A^2$ vs $1A^0$)**		
Mapk1	-2.32	0.023491
**Differentially Expressed Genes ($6A^2$ vs $1A^3$)**		
Ccr6	4.43	0.034447
Mx1	6.06	0.005957
Nfkb1	3.06	0.031248
Tlr3	6.94	0.016428
**Differentially Expressed Genes ($6A^4$ vs $1A^0$)**		
Cd86	-8.54	0.030853
Ticam1	-5.56	0.007244
**Differentially Expressed Genes ($6A^4$ vs $1A^3$)**		
Gata3	11.67	0.033026
Il18	11.00	0.034283
Irf3	2.36	0.020149
Lyz2	7.80	0.000772
Stat4	7.09	0.031649
**Differentially Expressed Genes ($1A^0$ vs $1A^3$)**		
Cd86	8.37	0.039116
Ddx58	2.37	0.016583
Fasl	5.44	0.012656
Il18	6.62	0.003422
Il1r1	11.77	0.020857
Irak1	5.99	0.000372
Ly96	6.50	0.025717
Mapk1	3.20	0.016987
Mapk8	4.15	0.010562
Nfkb1	4.32	0.026328
Stat4	9.48	0.025254
Ticam1	4.16	0.014790
Tlr7	18.08	0.010077
Tlr8	49.47	0.012963
Gapdh	4.73	0.005095
Stat1	-2.40	0.045731

References

1. Wang G, Guo X, Diangelo S, Thomas NJ, Floros J. Humanized SFTPA1 and SFTPA2 transgenic mice reveal functional divergence of SP-A1 and SP-A2: formation of tubular myelin in vivo requires both gene products. *J Biol Chem* 2010; 285: 11998-12010.
2. Oladunni FS, Park JG, Pino PA, Gonzalez O, Akhter A, Allué-Guardia A, Olmo-Fontánez A, Gautam S, Garcia-Vilanova A, Ye C, Chiem K, Headley C, Dwivedi V, Parodi LM, Alfson KJ, Staples HM, Schami A, Garcia JI, Whigham A, Platt RN, 2nd, Gazi M, Martinez J, Chuba C, Earley S, Rodriguez OH, Mdaki SD, Kavelish KN, Escalona R, Hallam CRA, Christie C, Patterson JL, Anderson TJC, Carrion R, Jr., Dick EJ, Jr., Hall-Ursone S, Schlesinger LS, Alvarez X, Kaushal D, Giavedoni LD, Turner J, Martinez-Sobrido L, Torrelles JB. Lethality of SARS-CoV-2 infection in K18 human angiotensin-converting enzyme 2 transgenic mice. *Nat Commun* 2020; 11: 6122.
3. Matute-Bello G, Downey G, Moore BB, Groshong SD, Matthay MA, Slutsky AS, Kuebler WM, Acute Lung Injury in Animals Study G. An official American Thoracic Society workshop report: features and measurements of experimental acute lung injury in animals. *Am J Respir Cell Mol Biol* 2011; 44: 725-738.
